# Taxonomic notes on Cyperaceae of Nepal: new records of a genus, six species and other noteworthy species

**DOI:** 10.3897/phytokeys.180.67634

**Published:** 2021-08-09

**Authors:** Prabin Bhandari, Satyam Chaudhary, Ajay Neupane, Shi-Liang Zhou, Shu-Ren Zhang

**Affiliations:** 1 State Key Laboratory of Systematic and Evolutionary Botany, Institute of Botany, Chinese Academy of Sciences, Beijing, 100093, China Institute of Botany, Chinese Academy of Sciences Beijing China; 2 University of Chinese Academy of Sciences, Beijing, 100049, China University of Chinese Academy of Sciences Beijing China; 3 Central Department of Environmental Sciences, Tribhuvan University, Kirtipur, Nepal Tribhuvan University Kirtipur Nepal; 4 Mechi Multiple Campus, Tribhuvan University, Bhadrapur, Jhapa, Nepal Tribhuvan University Bhadrapur Nepal

**Keywords:** *
Actinoscirpus grossus
*, *
Eleocharis ochrostachys
*, *
Fimbristylis
*, Flora of Nepal, *
Machaerina rubiginosa
*, *
Scleria rugosa
*, Tarai, wetland

## Abstract

This paper reports on the presence of one generic and six specific new records of Cyperaceous species for the flora of Nepal. Amongst the new discoveries are the genus *Machaerina*, alongside species: *Eleocharisochrostachys*, *Fimbristylisacuminata*, *F.ferruginea*, *F.nutans*, *F.thomsonii* and *Scleriarugosa*. The taxonomy and distribution of *Actinoscirpusgrossus*, *Fimbristylissalbundia* and *Fuirenaumbellata* in Nepal are clarified through notes on nomenclature, description, distribution, specimen examination, identification keys and photographs.

## Introduction

The sedge family, Cyperaceae, consisting of 95 genera and > 5600 species (Larridon et al. 2021) are predominantly perennial or annual herbs and are cosmopolitan in distribution (Goetghebeur 1998; Dai et al. 2010). Cyperaceae often have rhizomes and are distinguished by florets arranged in a spikelet, with a mostly triangular culm (stem). The ovary is superior and unilocular, producing an achene fruit, from anemophilous or entomophilous pollination (Goetghebeur 1998; Dai et al. 2010; Larridon et al. 2021).

In Nepal, Cyperaceae have a distribution range from tropical Tarai to alpine Himalaya (Rajbhandari and Rai 2017; Shrestha et al. 2018). So far, 213 species in 17 genera have been reported in Nepal (Rajbhandari and Rai 2017; Shrestha et al. 2018). However, literary research has indicated this is an incomplete record, as the South and South-East Asian genus *Machaerina* Vahl and some species in *Eleocharis* R.Br., *Fimbristylis* Vahl and *Scleria* P.J.Bergius remain absent from published works (Koyama 1978; Press et al. 2000; Rajbhandari and Baral 2010; Rajbhandari and Rai 2017; Shrestha et al. 2018). Furthermore, the occurrence of Actinoscirpusgrossus(L.f.)Goeth. & D.A.Simpsonvar.grossus, *Fimbristylissalbundia* (Nees) Kunth and *Fuirenaumbellata* Rottb. in Nepal is yet to be clarified (Clarke 1907; Halder and Dey 2016; Rajbhandari and Rai 2017).

Compared to other families, collections of Cyperaceae in Nepal are rather lacking. Plant exploration in Nepal is typically focused around mid-hills and high Himalayas; while the Tarai (lowlands) are mostly ignored (Rajbhandari 2016). To fill this gap, a series of field surveys was organised in both lowland and the valleys of mid-hills in Nepal from July 2019 to February 2021. Identification of the collected specimens revealed several new records for the flora of Nepal, including one genus and six species. The taxonomic status and distribution of *Actinoscirpusgrossus*, *Fimbristylissalbundia* and *Fuirenaumbellata* were also clarified.

## Methods

### Specimen collection and identification

Plant explorations were made in Tarai and valleys of mid-hills representing Western, Central and Eastern Nepal between July 2019 and February 2021. Wetlands around these regions were frequently visited and fruiting samples were collected. Fruiting individuals were collected, pressed, dried, mounted and deposited at the National Herbarium and Plant Laboratories (**KATH**) and Tribhuvan University Central Herbarium (**TUCH**). Field images of the living plant were captured with a Nikon D810 camera with lens attachment AF-S Micro Nikkor 105 mm.

The specimens were identified with reference to literature (Kern 1974; Rao and Verma 1982; Noltie 1994; Dai et al. 2010; Dey and Prasanna 2015; Simpson 2019) and comparing them to the specimens in various herbaria. KATH and TUCH were visited and utilised to examine the dried samples, while the digital images were accessed through online databases of BM, E, K and TI (acronyms following Thiers 2021 onwards). The culm, leaf and achene morphologies were observed under a zoom stereomicroscope ZSM-111.

## Results

### Taxonomic treatment

#### *Actinoscirpus* (Ohwi) R.W.Haines & Lye, Bot. Not. 124: 481. 1971.

##### Actinoscirpus grossus

Taxon classificationPlantaePoalesCyperaceae

(L.f.) Goetgh. & D.A.Simpson, Kew Bull. 46(1): 171. 1991.

F06B90AB-559B-53E1-BBCC-6BDA747239B5

Scirpus grossus L.f., Suppl. Pl. 104. 1782.
Schoenoplectus grossus (L.f.) Palla, Allg. Bot. Z. Syst. 3. 1911.

###### Type.

India, collector unknown s.n. [lectotype, designated by Goetghebeur and Simpson 1991, pg. 171: LINN (Herb no. 71/32 image!)].

###### Description.

Perennial herbs, stolon bearing. Culm up to 2 m high, acutely 3-angled, smooth or scabrid. Leaves linear, 8–19 mm wide, margin entire to scabrid. Involucral bracts leaf-like exceeding inflorescence to 60 cm. Inflorescence in terminal anthela bearing many spikelets. Spikelets ovoid-ellipsoid, 2–4 × 2–3 mm. Glumes broadly ovate or elliptic to oblong, brownish, 2–3 × 1.5–2.5 mm, membranous, abaxially pubescent, margin ciliate, apex apiculate or mucronate, single-veined. Perianth bristles 6, retrorsely scabrous or plumose, slightly shorter to slightly longer than achene. Stamens 3, longer than achene. Anthers 1.5 mm. Style 1 mm long. Stigmas 3, 1 mm long. Achene 3-sided, obovoid, 1.5 × 1–1.3 mm, brownish, smooth.

### Key to the varieties

**Table:** 

1a	Culm angle smooth, glumes apiculate, perianth bristles retrorsely scabrous	** var. grossus **
1b	Culm angle scabrid, glumes mucronate, perianth bristles plumose	** var. kysoor **

#### Actinoscirpus grossus var. grossus

Taxon classificationPlantaePoalesCyperaceae

73E88095-53CB-5789-B8F9-948BE6E0AD49

Actinoscirpus grossus var. kysoor auct. non (Roxb.) Noltie, in Handb. Fl. Pl. Nepal 1: 206. 2017, (R.R. Kafle 7, (TUCH))

##### Description.

**Figure 1.**
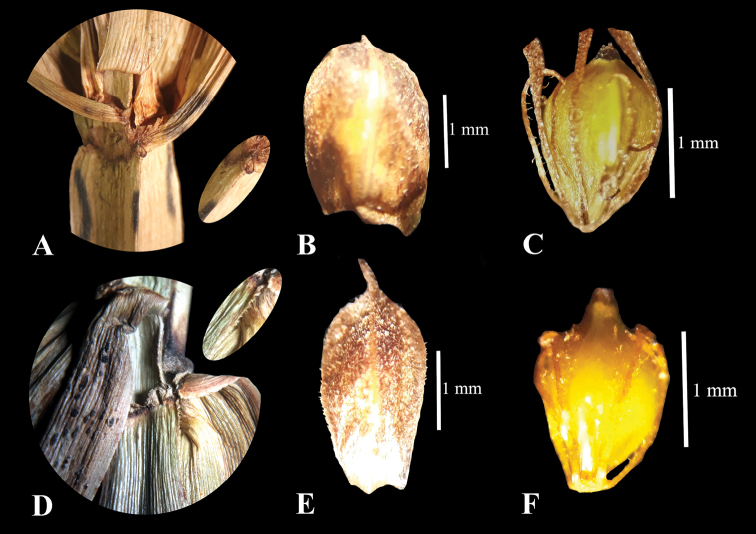
Actinoscirpusgrossusvar.grossus**A** culm **B** glume **C** achene; A.grossusvar.kysoor**D** culm **E** glume **F** achene.

Culm angle entire. Glumes apiculate. Perianth bristles retrorsely scabrous. (Fig. 1A–C).

##### Nepali name.

Gudh, Gulgulia

##### Distribution.

Nepal, India, Bhutan, China, Myanmar, Bangladesh, Sri Lanka, Pakistan, Laos, Vietnam, Malaysia, Thailand, Indonesia, Philippines, New Guinea and Australia.

##### Ecology.

Grows in paddy field and lake banks; 600–630 m elev.

##### Phenology.

Flowering in July–September; fruiting in October–December.

##### Specimens examined.

**Nepal, Kaski**: Pokhara Valley, Rupa Lake, 28°9'54.31"N, 84°7'24.48"E, 630 m elev., 17 Sep 2019, *P. Bhandari & V. Adhikari KAS07* (KATH); **Kaski**: Pokhara Valley, Rupa Lake, 600 m elev. 15 Apr 1999, *R.R. Kafle 7* (TUCH); **Nawalparasi**: 05 Aug 2007, *S. Dahal 20074* (KATH).

#### Actinoscirpus grossus var. kysoor

Taxon classificationPlantaePoalesCyperaceae

(Roxb.) Noltie, Edinburgh J. Bot. 51(2): 173. 1994.

DB6800F2-19C9-5B89-B5FA-98EDD123F1DA

Scirpus kysoor Roxb., Fl. Ind. 1: 235. 1820.
Scirpus grossus var. kysoor (Roxb.) Clarke, Fl. Brit. India 6: 660. 1894.
Scirpus grossus f. kysoor (Roxb.) Beetle, Amer. J. Bot. 33(8): 661. 1946.
Schoenoplectus grossus auct. non (L.f.) Palla, in Enum. Pl. Nepal 1: 118. 1978.

##### Type.

Roxburgh Icones No. 2017 [(lectotype, designated by Noltie 1994, pg. 173: K, n.v.), (epitype, designated by Noltie 1994, pg. 173: E (E00386664 image!))].

##### Description.

Culm angle scabrous towards the apex. Glume with a distinct 0.5 mm, recurved mucro. Perianth bristles plumose. (Fig. 1D–F).

##### Distribution.

Nepal, India and Bhutan.

##### Ecology.

Grows in paddy fields; 71 m elev.

##### Phenology.

Flowering in August–September; fruiting in October–December.

##### Specimen examined.

**Nepal, Jhapa**: Kachankawal Rural Municipality (RM), Baniyani, 26°26'15.94"N, 88°3'1.28"E, 71 m elev., 04 Dec 2020, *S. Chaudhary 20120410* (KATH, TUCH).

##### Note.

The first author visited: Rupa Lake and other wetlands of Pokhara Valley, Central Nepal and Jhapa District, East Nepal; observing multiple specimens. Samples deposited at KATH (Central Nepal, *S. Dahal 20074*) and TUCH (Central Nepal, *R.R. Kafle 7*) were examined. Upon close inspection of the culm, glume and achene characters, the Central Nepal populations exactly match Actinoscirpusgrossusvar.grossus, while the East Nepal populations matched with that of Actinoscirpusgrossusvar.kysoor. Therefore, it can be concluded that the two varieties of *Actinoscirpusgrossus* occur in Nepal.

###### *Eleocharis* R.Br., Prodr. Fl. Nov. Holland. 224. 1810.

#### Eleocharis ochrostachys

Taxon classificationPlantaePoalesCyperaceae

Steud., Syn. Pl. Glumac. 2(7): 80. 1855.

C693F411-ED15-57CE-A11C-643031CB07E4

Scirpus ochrostachys (Steud.) Kuntze, Revis. Gen. Pl. 2: 758. 1891.

##### Type.

Indonesia, Java, 17 August 1842, *H. Zollinger 291* [holotype: P (P00329735 image!)].

##### Description.

**Figure 2.**
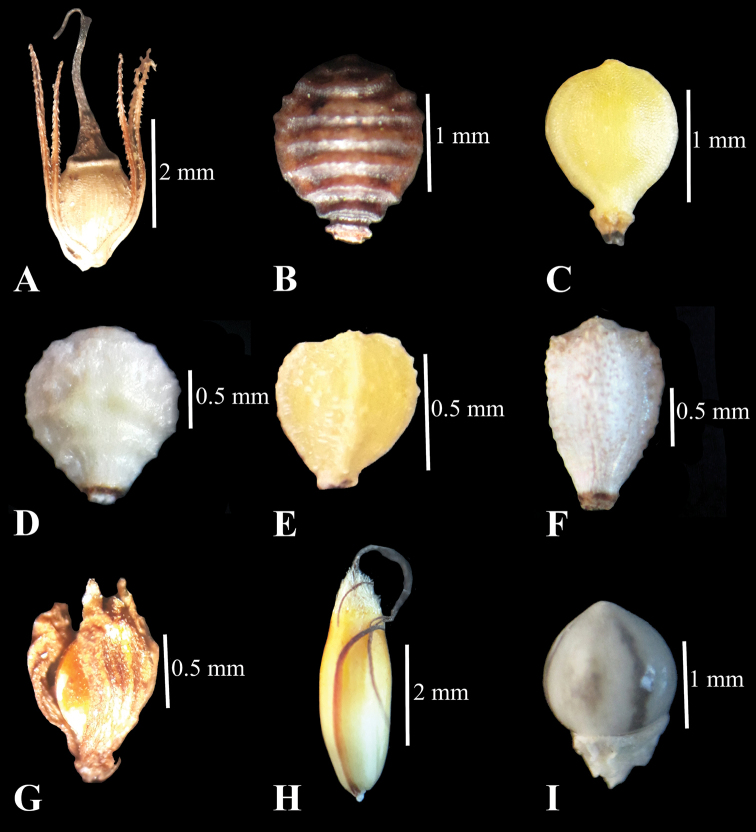
Achene **A***Eleocharisochrostachys***B***Fimbristylisacuminata***C***F.ferruginea***D***F.nutans***E***F.salbundia***F***F.thomsonii***G***Fuirenaumbellata***H***Machaerinarubiginosa***I***Scleriarugosa*.

Plant 30–110 cm tall, stoloniferous, tufted. Sheaths 2, tubular, purplish-red to pale green or hyaline, 4–12 cm, mouth obliquely truncate, apex acute. Culm round, sometimes obscurely 3–angled, lacking septa. Spikelet cylindrical to ovoid, 0.7–3.2 cm with many spirally arranged glumes; lowermost glume empty. Glume ovate, leathery, 3.5–4.5 × 2–3.5 mm, margin hyaline, apex obtuse. Persistent style base flattened, up to half the width of achene. Stigmas 2 or 3. Stamens 3, as long as perianth bristles; anther 2 mm. Perianth bristles 6 to 8, almost twice or more than twice the length of achene, retrorsely scabrous. Achene biconvex, obovoid, 1.5–2 × 1.2–1.5 mm, yellowish-brownish, shiny, surface longitudinally striate with more than 25 rows of transversely linear-oblong epidermal cells, apex with an annular thickening, forming a small neck. (Fig. 2A).

##### Distribution.

Nepal (new record), India, China, Myanmar, Bangladesh, Sri Lanka, Malaysia, Thailand, Indonesia, Philippines, Vietnam and New Guinea.

##### Ecology.

Grows in marshy areas, floating islands, lake edges and waterlogged fields; 70–762 m elev.

##### Phenology.

Flowering in August-September; fruiting in October–December.

##### Specimens examined.

**Nepal, Kaski**: Pokhara valley, Begnas Lake, 690 m elev., 17 September 2019, *P. Bhandari & V. Adhikari KAS14* (KATH); **Kaski**: Pokhara Valley, Gunde Lake, 28°11'30.29"N, 84°2'21.58"E, 762 m elev., 09 Dec 2020, *P. Bhandari & V. Thapa 20120912* (KATH); **Kaski**: Pokhara Valley, Neureni Lake, 28°11'30.37"N, 84°2'52.30"E, 749 m elev., 30 Dec 2020, *P. Bhandari & N.L. Bhandari 20123002* (KATH); **Jhapa**: Kachankawal RM, Aambari Road, Thulo Kechana, 26°25'36.56"N, 87°59'15.75"E, 70 m elev., 05 Feb 2021, *P. Bhandari*, *A. Neupane & S. Chaudhary 21020505* (KATH).

### Keys to the species of *Eleocharis* in Nepal

**Table:** 

1a	Spikelet usually cylindrical or narrowly ellipsoid as wide as culms	**2**
1b	Spikelet ovoid to narrowly ovoid, rarely cylindrical usually wider than culm	**4**
2a	Culms with transverse septa, spikelet with basal two glumes empty, achene smooth	*** E. dulcis ***
2b	Culms without transverse septa, spikelet with only basal-most glume empty, achene surface cancellate or reticulate	**3**
3a	Culms terete, sometimes weakly 3-angled; achene apex lacking constriction	*** E. ochrostachys ***
3b	Culms sharply 3-angled, achene apex with a distinct constriction	*** E. acutangula ***
4a	Persistent style base scarcely differentiated from achene apex and seemingly a continuation of it	**5**
4b	Persistent style base differentiated from achene apex by a constriction or articulation at the junction with it	**6**
5a	Plant annual, achene winged or angled, reticulate to deeply pitted-reticulate with isodiametric epidermal cells	*** E. retroflexa ***
5b	Plant perennial, not winged or angled, smooth	*** E. quinquiflora ***
6a	Stigmas 3	**7**
6b	Stigmas 2	**8**
7a	Culms acutely angled, spikelet not proliferous	*** E. tetraquetra ***
7b	Culms terete, spikelet usually proliferous at base	*** E. congesta ***
8a	Annual, persistent style base not spongy thickened	*** E. atropurpurea ***
8b	Perennial, persistent style base spongy thickened	**9**
9a	Only lowermost glumes empty, surrounding the spikelet base completely or 3/4 of it	*** E. uniglumis ***
9b	2 or 3 basal glumes empty, the lowermost glume surrounds about 2/3 of the spikelet base	*** E. palustris ***

#### *Fimbristylis* Vahl, Enum. Pl. 2: 285. 1805.

##### Fimbristylis acuminata

Taxon classificationPlantaePoalesCyperaceae

Vahl, Enum. Pl. 2: 285. 1805.

C0503B23-C8D5-5583-B9EC-6A6F026AC583

###### Type.

India, *König* s.n. [holotype: C, (C10010413 image!)].

###### Description.

Plant annual, tufted. Culm terete, up to 22 cm long. Leaf reduced to the bladeless sheath. Involucral bract absent. Inflorescence terminal with a single erect or slightly nodding spikelet. Spikelet lanceolate-ovoid, 5–10 × 1.5–3 mm. Glumes pale green or brown-tinged, ovate, 3–4 × 2–2.5 mm, apex obtuse and mucronate. Style flattened, apically ciliate, 2.5 mm long. Stigmas 2, ciliate. Stamens 2, as long as glumes. Achene brownish-black, obovoid, 1.5 × 1–1.5 mm, biconvex, with 6–7 rows of transversely wavy reticulation in each face, pitted with hexagonal cells, shortly stipitate. (Fig. 2B).

###### Distribution.

Nepal (new record), Bhutan, India, China, Bangladesh, Sri Lanka, Laos, Malaysia, Thailand, Indonesia, Philippines, Vietnam, New Guinea and Australia.

###### Ecology.

Grows in flood plains, riverbanks and semi-dry paddy fields; 70–676 m elev.

###### Phenology.

Flowering in August-September; fruiting in October–December.

###### Specimens examined.

**Nepal, Kaski**: Pokhara Valley, Sita Paila, 28°10'30.92"N, 83°59'40.36"E, 676 m elev., 11 Oct 2020, *P. Bhandari & A. Bhandari 20101101* (KATH, TUCH); **Jhapa**: Kachankawal RM, Baniyani, 26°26'15.94"N, 88°3'1.28"E, 70 m elev., 04 Dec 2020, *S. Chaudhary 20120406* (KATH).

##### Fimbristylis ferruginea

Taxon classificationPlantaePoalesCyperaceae

(L.) Vahl, Enum. Pl. 2: 291. 1805.

1DD910BF-6E67-54F2-9ED7-ADD64458A3C2

Scirpus ferrugineus L., Sp. Pl. 1: 50. 1753.

###### Type.

Jamaica, Collector unknown s.n. [Herb. van Royen], [lectotype, designated by Adams in Cafferty and Jarvis 2004, pg. 180: L, (L0052731 image!)].

###### Description.

Plant tufted, 20–50 cm tall. Culm many, irregularly angled, bladeless sheath present. Leaves shorter than culm, ligulate, 1.5–3 mm wide, margin scabrous. Involucral bracts overtopping spikelet, to 9.5 cm long. Inflorescence terminal with 4–6 spikelets, sometimes with one or two spikelets. Spikelets ovoid, 5–10 × 2–4 mm. Glumes broadly ovoid, 3–3.5 × 2.5–3 mm, puberulous apically, apiculate, vein single. Style dorsoventrally flattened, 1–1.5 mm long, apically ciliate. Stigmas 2, ciliate, slightly shorter than style. Stamens 3, double the length of the achene. Achene obovoid, biconvex, creamy, 1.2–1.5 × 1 mm, surface smooth, obscurely pitted with hexagonal cells, distinctly stipitate. (Fig. 2C).

###### Distribution.

Nepal (new record) and pantropical.

###### Ecology.

Grows in flood plains and riverbanks; 676–684 m elev.

###### Phenology.

Flowering in July–August; fruiting in September–December.

###### Specimens examined.

**Nepal, Kaski**: Pokhara Valley, Sita Paila, 28°10'34.29"N, 83°59'42.20"E, 684 m elev., 27 Jun 2020, *P. Bhandari*, *R. Chapagain & A. Bhandari 20062704* (KATH); Pokhara Valley, Sita Paila, 28°10'30.92"N, 83°59'40.36"E, 676 m elev., 11 Oct 2020, *P. Bhandari & A. Bhandari 20101102* (KATH); Pokhara Valley, Sita Paila, 28°10'34.29"N, 83°59'42.20"E, 684 m elev., 21 Dec 2020, *P. Bhandari 20122103* (KATH).

###### Note.

A few populations were observed with one or two spikelets, emulating a pseudo-lateral inflorescence appearance.

##### Fimbristylis nutans

Taxon classificationPlantaePoalesCyperaceae

(Retz.) Vahl, Enum. Pl. 2: 285. 1805.

CC9FA5DD-4D71-5F49-9B55-2CC869C8384C

Scirpus nutans Retz., Observ. Bot. 4: 12. 1786.

###### Type.

Malaysia, Malacca, *J.G. König* s.n. [lectotype, designated by Fischer 1932, pg. 69: LD (LD1283267 image!)].

###### Description.

Perennial tufted herb. Leaves reduced to a tubular, bladeless sheath. Involucral bract glume-like, 3.5 mm long. Inflorescence consisting of a single terminal spikelet. Spikelet slightly nodding, ovoid with spirally arranged glumes. Glumes 2–4 × 1.5–3 mm, rust-brown, oblong-elliptic, margin membranous, apiculate. Style 3.5 mm long, flattened with ciliate margin. Stigmas 2. Stamens as long as style. Achene white, obovate, 1.5 × 1 mm, biconvex, with transverse wavy reticulation, basal stipe indistinct. (Fig. 2D).

###### Distribution.

Nepal (new record), India, China, Myanmar, Malaysia, Indonesia, Thailand, Papua New Guinea, Sir Lanka, Vietnam and Australia.

###### Ecology.

Grows in marshy areas, edges of the lake; sometimes forming a floating island of vegetation, associated with *Eleocharis* species and *Fimbristylis* species; 700 m elev.

###### Phenology.

Flowering in July–August; fruiting in September–October.

###### Specimen examined.

**Nepal, Kaski**: Pokhara Valley, Dipang Lake, 28°10'57.90"N, 84° 4'9.28"E, 700 m elev., 17 Sep 2019, *P. Bhandari & V. Adhikari KAS13* (KATH).

##### Fimbristylis salbundia

Taxon classificationPlantaePoalesCyperaceae

(Nees) Kunth, Enum. Pl. 2: 230. 1837.

7FB6CFD4-5E29-5D8C-8C71-847A3B8D3957

Trichelostylis salbundia Nees, Contr. Bot. India 105. 1834.

###### Type.

India, Silhet, *N. Wallich* 3526 [lectotype, designated by Halder and Dey 2016, pg. 357, 359: K (K000974061 image!)].

###### Description.

Plant rhizomatous, not tufted. Culm up to 130 cm, 5-angled. Leaf reduced to the bladeless sheath, up to 18 cm, tubular. Involucral bracts setaceous to 1 cm long. Inflorescence a compound anthela. Spikelet ovoid, 3.5–4 × 1.5–2 mm, with spirally arranged glumes. Glumes elliptic-ovoid, 1.8–2 × 1 mm, middle part chestnut brown, margin membranous, 3–veined, apex obtuse to acute, not mucronate. Style 1 mm, trigonal, basally inflated, not ciliate. Stigmas 3, as long as style, plumose. Stamens 3, 2 mm long. Achene obovoid, trigonal, 0.5–0.7 × 0.5 mm, sparsely verruculose with transversely oblong epidermal cells in more than 9 vertical rows on each face. (Fig. 2E).

###### Distribution.

Nepal, India, China, Myanmar, Bangladesh, Sri Lanka, Philippines, Vietnam, Thailand, Indonesia and New Guinea.

###### Ecology.

Grows in marshy areas; 760–835 m elev.

###### Phenology.

Flowering in July–September; fruiting in October–December.

###### Specimens examined.

**Nepal, Dang**: Tulsipur, near Damargau, Angare, 835 m elev., 17 Dec 2020, *B. Subedi 20121704* (KATH); **Kaski**: Pokhara Valley, Gunde Lake, 28°11'30.29"N, 84°2'21.58"E, 760 m elev., 30 Dec 2020, *P. Bhandari & N.L. Bhandari 20123005* (KATH, TUCH).

###### Note.

The protologue of *Fimbristylissalbundia* [≡ *Trichelostylissalbundia*] was based on two collections of Wallich, i.e. *Wallich 3499* and *3526* from ‘Nepalia’ and ‘Silhet’, respectively (Wallich 1828; Nees 1834). All collections representing *3499* were later annotated as *F.quinquangularis* (Vahl) Kunth., except *3499c* at B, which was *F.salbundia* (Nees) Kunth (Clarke 1907). The collection *3499c* at B was destroyed in 1943, during the Second World War (Halder and Dey 2016). Subsequently, the occurrence of *F.salbundia* was not reported in the published works (Koyama 1978; Press et al. 2000; Rajbhandari and Rai 2017; Shrestha et al. 2018; POWO 2019; Govaerts et al. 2021). The rediscovery of *F.salbundia* after 200 years confirms the occurrence of this taxon in Nepal.

*Fimbristylissalbundia* is very similar to *F.quinquangularis*, but can be distinguished, based on the nature of its leaf sheaths and achene character. *Fimbristylissalbundia* is characterised by the presence of bladeless sheaths and sparsely verruculose achene, surface pitted with more than nine vertical rows of transversely oblong epidermal cells. However, *Fimbristylisquinquangularis* has leaf sheaths with blades and densely verruculose achene with up to six vertical rows of transversely linear-oblong epidermal cells.

##### Fimbristylis thomsonii

Taxon classificationPlantaePoalesCyperaceae

Boeckeler, Linnaea 37(1): 37. 1871.

C4ADDF3E-ED4A-5A3C-A660-5567B1844984

###### Type.

India, Mount Khasia, *J.D. Hooker & T. Thomson* 12 [lectotype, designated by Dey and Halder 2015, pg. 230, 231: P (P00051618 image!)].

###### Description.

Plant more than 20 cm. Blade less sheath lacking. Leaf-blade flat, 2 mm wide, margin scabrous, ligulate. Involucral bract shorter than inflorescence, to 4.5 cm. Inflorescence terminal in compound anthela with more than 20 spikelets. Spikelets 5–7 × 1.5–3 mm, elliptic, ovoid to oblong, reddish. Glumes boat-shaped, ovate, chestnut brown, 3–3.5 × 2 mm, mid-vein keeled, arising from the base and excurrent into a mucro, 3 more lines arising each side of mid-vein from base to apex, surface glabrous, margin membranous. Style 1.5 mm long, base inflated, 3-angled, not ciliate. Stigmas 3. Stamens 3, 3 mm long. Achene white, shiny, trigonous, obovate, 1.5 × 1 mm, verruculose. (Fig. 2F).

###### Distribution.

Nepal (new record), India, China, Myanmar, Bangladesh, Laos, Malaysia, Philippines, Vietnam and Thailand.

###### Ecology.

Grows in grassland, near *Schima-Castanopsis* forest; 1020 m elev.

###### Phenology.

Flowering and fruiting in April.

###### Specimen examined.

**Nepal, Kaski**: Pokhara, Kharchyang-Aghihare Community Forest, Bhirswara, 28°9'34.27"N, 83°59'32.80"E, 1020 m elev., 25 Apr 2020, *P. Bhandari & A. Bhandari KAS28* (KATH).

### Keys to the species of *Fimbristylis* in Nepal

**Table:** 

1a	Stigmas 3	**2**
1b	Stigmas 2	**12**
2a	Glumes distichous at least in the lower part of spikelet	**3**
2b	Glumes spirally arranged	**5**
3a	Inflorescence reduced to single spikelets; involucral bract glume-like	*** F. ovata ***
3b	Inflorescence with more than two spikelets; involucral bract setaceous or foliaceous	**4**
4a	Perennials; involucral bract foliaceous; inflorescence a compound anthela; glumes glabrous	*** F. fusca ***
4b	Annual; involucral bract setaceous; inflorescence a simple or sub-compound anthela; glumes apically sparsely ciliate	*** F. fimbristyloides ***
5a	Leaf-sheath ligulate, with a fringe of short hairs	**6**
5b	Leaf-sheath eligulate	**7**
6a	Stem strongly compressed; involucral bract exceeding inflorescence; achene smooth	*** F. complanata ***
6b	Stem not compressed; involucral bract shorter than inflorescence; achene verruculose	*** F. thomsonii ***
7a	Spikelets in clusters	*** F. falcata ***
7b	Spikelets solitary	**8**
8a	Culm 3-angled	**9**
8b	Culm 4 or 5-angled	**10**
9a	Plant stoloniferous; culm with all leaves with a blade	*** F. pierotii ***
9b	Plant not stoloniferous; culms with 1–3 leafless sheaths	*** F. umbellaris ***
10a	Culm 4-angled; leaf blade bilaterally flattened, ensiform; spikelet globose	*** F. littoralis ***
10b	Culm 5-angled; leaf blade (if present) dorsoventrally flattened, linear; spikelets elongated	**11**
11a	Leaves with a blade; achene distinctly verruculose with 4–7 rows of transversely oblong epidermal cells	*** F. quinquangularis ***
11b	Leaves reduced to bladeless sheath; achene sparsely verruculose with more than 9 rows of transversely linear-oblong epidermal cells	*** F. salbundia ***
12a	Spikelets 1 to 3	**13**
12b	Spikelets several to many (sometimes, one or two spikelets in *F.ferruginea*, but glume is always apically puberulous)	**15**
13a	Leaves with a blade; achene smooth, pitted with hexagonal cells	*** F. schoenoides ***
13b	Leaves reduced to a bladeless sheath; achene coarsely rugulose with transverse wavy reticulation	**14**
14a	Involucral bracts glume-like; spikelet nodding; achene margin verruculose	*** F. nutans ***
14b	Involucral bract absent; spikelet erect; achene margin not verruculose	*** F. acuminata ***
15a	Leaves eligulate	**16**
15b	Leaves ligulate	**20**
16a	Annual	**17**
16b	Perennial	**18**
17a	Style base fringed with a whorl of long pendent hairs covering the upper half of the nut	*** F. squarrosa ***
17b	Style minutely ciliate at the top	*** F. aestivalis ***
18a	Rhizome creeping; culm sparsely tufted; spikelets not angular	*** F. rigidula ***
18b	Rhizome not creeping; culm densely tufted; spikelets slightly angular by the keeled glumes	**19**
19a	Leaves flat or canaliculate, apex abruptly acuminate; glumes keel glabrous	** F. cymosa var. spathacea **
19b	Leaves flat, apex subobtuse; glumes keel puberulous	*** F. fuscinux ***
20a	Plant bearing stolons	*** F. stolonifera ***
20b	Plant tufted, lacking stolons	**21**
21a	Glumes puberulous, broadly ovoid, margin apically ciliate, apiculate; achene smooth, obscurely pitted with hexagonal cells	*** F. ferruginea ***
22b	Glumes glabrous, margin hyaline, 3-veined, acute to apiculate; achene reticulate with transversely oblong cells	**22**
22a	Spikelets 1–1.5 mm wide, angular by the keeled glume; glumes to 1.5 mm long	*** F. bisumbellata ***
22b	Spikelets 2–4 mm wide, terete; glumes not keeled, over 1.5 mm long	*** F. dichotoma ***

#### *Fuirena* Rott., Descr. Icon. Rar. Pl. 70. 1773.

##### Fuirena umbellata

Taxon classificationPlantaePoalesCyperaceae

Rottb., Descr. Icon. Rar. Pl. 70, t. 19, f. 3. 1773.

7B5912BB-AF1E-52D5-9A25-FFB3E04F64A3

###### Type.

Suriname, *D. Rolander* s.n. (lectotype, designated by De Moraes 2012, pg. 65: SBT 1.3.1.47, image!).

###### Description.

Perennial rhizomatous herbs. Culm solitary, lowermost node swollen, 120 cm tall, acutely 5-angled, glabrous to puberulous. Leaves linear-lanceolate, 13–17 × 1.5–1.7 mm, 5-veined, apex acuminate, margin ciliate; ligule brownish, hyaline, truncate. Lower involucral bracts leaf-like, 2–10 cm long, sheath densely pubescent; upper bracts much shorter, not or hardly sheathing. Inflorescence with 8–14 glomerulate clusters of spikelets; glomerulate bearing 6–30 spikelets arising from a white villous pedicle. Spikelets greenish-brown, ovoid-ellipsoid, 4–7.5 × 2–3 mm. Glumes 2–2.5 × 1.5 mm, ellipsoid to oblong, membranous, blackish to brownish tinged, abaxially pilose towards the emarginated apex, 3-veined costa ending in a short puberulent, 0.7–1 mm awn. Perianth segments 6 in two whorls; outer 3, needle-like, whitish, apically scabrous, as long as or shorter than the stalk of inner bristles; inner bristles 3, obovoid to oblong, whitish to brownish, 1–1.5 × 0.5–0.7 mm, membranous, margin ciliate, apically densely ciliate, base gradually narrowed down into a twisted 0.5 mm stalk, apex emarginate, veins 3, ending in a short recurved awn. Stamens 3, 2.5 mm long. Anthers oblong, 0.8–1 mm long. Style 1 mm long. Stigmas 3, 1 mm long, plumose. Achene brown, ellipsoid to obovoid, 3-sided, 0.8 × 0.7–1 mm (including stipitate base), apex with a 3 mm punctate conical whitish beak. (Fig. 2G).

###### Distribution.

Nepal and pantropical.

###### Ecology.

Grows in marshy areas; 70–149 m elev.

###### Phenology.

Flowering and fruiting in January–March.

###### Specimens examined.

**Nepal, Jhapa**: Kachankawal RM, Aambari Road, Thulo Kechana, 26°25'36.56"N, 87°59'15.75"E, 70 m elev., 05 Feb 2021, *P. Bhandari*, *A. Neupane & S. Chaudhary 21020504* (KATH, TUCH); **Jhapa**: Salbadi, 26°40'26.75"N, 88°0'51.90"E, 149 m elev., 05 Feb 2021, *P. Bhandari*, *A. Neupane & S. Chaudhary 21020503* (KATH).

###### Note.

Published literature of Nepalese flora (Koyama 1978; Press et al. 2000; Rajbhandari and Baral 2010; Rajbhandari and Rai 2017; Shrestha et al. 2018) had not previously reported this species. However, the present finding supports Govaerts et al. (2021), which had reported its distribution in Nepal.

The Nepalese populations bear perianth segments in two whorls. The outer whorl consists of three very short needles like bristles, while the inner whorl consists of three obovate-oblong perianth segments.

### Keys to the species of *Fuirena* in Nepal

**Table:** 

1a	Perennial with short rhizome; basal node of culm swollen; inner perianth segments obovate, gradually narrowed at base	*** F. umbellata ***
1b	Annual lacking rhizome; culm lacking swollen structure; inner perianth segments subquadrate, abruptly narrowed at base	*** F. ciliaris ***

#### *Machaerina* Vahl, Enum. Pl. 2: 238. 1805.

##### Machaerina rubiginosa

Taxon classificationPlantaePoalesCyperaceae

(Biehler) T.Koyama, Bot. Mag. (Tokyo) 69(812): 65. 1956.

7ECBECFC-40D1-5587-8705-6335F082E22F

Fuirena rubiginosa Biehler, Pl. Nov. Herb. Spreng. 3. 1807.

###### Type.

New Zealand, *Forster* s.n. [lectotype, designated by Garnock-Jones 1986, pg. 125: K (K000883942 image!)].

###### Description.

Plant rhizomatous. Culms tufted, compressed to subterete, 0.7–1.2 m tall. Leaves shorter than or equalling the culm, blade biconvex with obtuse edges, apex acute. Inflorescence paniculate, laxly arranged. Spikelets ovoid, with two flowers. Glumes lanceolate, 5–6 × 2.5–3 mm, brownish, mid-vein keeled, lower part greenish, margin ciliate, apex acuminate. Style base densely sericeous. Style short, to 1.5 mm. Stigmas 3, to 6 mm long. Stamens 3, longer than achene. Anther 2.5 mm long. Achene trigonous, ellipsoid, yellowish-orange, shiny, 4–5 × 1.5–1.8 mm. (Fig. 2H).

###### Nepali name.

Gudh

###### Use.

Culm is collected to weave handmade mats (locally called ‘gundri’).

###### Distribution.

Nepal (new record), India, China, Bangladesh, Sri Lanka, Vietnam, Malaysia, Indonesia, Philippines, New Guinea and Australia.

###### Ecology.

Grows in the floating island and marshy areas; 762 m elev.

###### Phenology.

Flowering in July–September; fruiting in December–January.

###### Specimen examined.

**Nepal, Kaski**: Pokhara Valley, Gunde Lake, 28°11'30.29"N, 84°2'21.58"E, 762 m elev., 09 Dec 2020, *P. Bhandari* & *V. Thapa 20120913* (KATH, TUCH).

###### Note.

This is the first report of this genus in Nepal. The genus *Machaerina* shows morphological similarities to the genera *Cladium* and *Rhynchospora*. *Cladium* is differentiated by its solid stem and ellipsoid achene lacking disc. *Machaerina* is differentiated from *Rhynchospora* by its leaf characters; the leaves in *Machaerina* are distichously arranged, whereas in *Rhynchospora*, they are tristichously arranged.

#### *Scleria* P.J.Bergius, Kongl. Vetensk. Acad. Handl. 26: 142 (–144) 1765.

##### Scleria rugosa

Taxon classificationPlantaePoalesCyperaceae

R.Br., Prodr. Fl. Nov. Holland. 240. 1810.

3699366D-90E7-5960-A72C-D937CED1CB64

###### Type.

Australia, Queensland, Endeavour River, 1770, *J. Banks* & *D. Solander* s.n. [lectotype, designated by Simpson 2019, pg. 207: BM (BM000833641 image!)].

###### Description.

Plant annual. Stem suberect, culm tufted, glabrous, 3-angled, to 10 cm long. Basal sheath glabrous, leaf sheath sparsely ciliate, not winged; contraligule round, barbate. Leaf-blade linear, glabrous, 2.5–7 × 0.25–3 cm. Involucral bracts leaf-like, to 11 cm long, glabrous. Inflorescence paniculate with 1–3 distant branches, each branch, with 1–3 spikelets. Peduncle recurved, slightly winged, margin ciliate. Spikelets unisexual; male spikelets linear-lanceolate, shortly peduncled, margin ciliate, glumes to 2 mm long, lanceolate, the mid-vein of the two outer ones ciliate; female spikelets with up to 5 glumes. Glumes ovate-lanceolate, 2–4 × 1–1.5 mm, beset with long, patent hairs, vein prolonged into a mucro. Achene spherical-globose, 1.5 × 1.5 mm, whitish or greyish, smooth, shiny, apex with a whitish beak. Disc thick, lobe yellowish-brown, shallowly 3-lobed, rounded-obtuse, margin reflexed. (Fig. 2I).

###### Distribution.

Nepal (new record), India, China, Myanmar, Bangladesh, Sri Lanka, Malaysia, Thailand, Indonesia, Philippines, Vietnam, New Guinea, Japan, Korea and Australia.

###### Ecology.

Understorey of *Schima*-*Castanopsis* forest and edges of water canal; 71–755 m elev.

###### Phenology.

Flowering in August–September; fruiting in October–December.

###### Specimens examined.

**Nepal, Kaski**: Pokhara Valley, Niureni Lake, 28°11'32.96"N, 84°2'53.63"E, 755 m elev., 08 Sep 2020, *P. Bhandari 20090802* (KATH); **Kaski**: Pokhara Valley, Niureni Lake, 28°11'32.96"N, 84°2'53.63"E, 749 m elev., 30 Dec 2020, *P. Bhandari* & *N.L. Bhandari 20123007* (KATH); **Jhapa**: Kachankawal RM, Baniyani, 26°26'15.94"N, 88°3'1.28"E, 71 m elev., 04 Dec 2020, *S. Chaudhary 20120410* (KATH).

### Keys to the species of *Scleria* in Nepal

**Table:** 

1a	Annual, rhizome absent	**2**
1b	Perennial, rhizome present	**5**
2a	Glumes beset with long, patent hairs; disc shallowly lobed	*** S. rugosa ***
2b	Glumes glabrous; disc obsolete or tri-lobed	**3**
3a	Inflorescence spiciform, unbranched, lacking leafy bracts; disc obsolete	*** S. pergracilis ***
3b	Inflorescence paniculate, having a terminal and lateral panicles, leaf bracts present; disc tri-lobed	**4**
4a	Achene spherical with dark purplish beak, deeply pitted; disc lobe acuminate at apex	*** S. biflora ***
4b	Achene ellipsoid or subglobose with white beak, not deeply pitted; disc lobe acute at apex	*** S. parvula ***
5a	Plant much robust; achene cancellate; disc lobe obtuse or rounded at apex	*** S. terrestris ***
5b	Plant smaller; achene smooth or slightly rugulose; disc lobe acute at apex, often bidentate	*** S. levis ***

## Supplementary Material

XML Treatment for Actinoscirpus grossus

XML Treatment for Actinoscirpus grossus var. grossus

XML Treatment for Actinoscirpus grossus var. kysoor

XML Treatment for Eleocharis ochrostachys

XML Treatment for Fimbristylis acuminata

XML Treatment for Fimbristylis ferruginea

XML Treatment for Fimbristylis nutans

XML Treatment for Fimbristylis salbundia

XML Treatment for Fimbristylis thomsonii

XML Treatment for Fuirena umbellata

XML Treatment for Machaerina rubiginosa

XML Treatment for Scleria rugosa
